# On the Practical Advantages of the Use of Flannel Worn Next the Skin

**Published:** 1799-03

**Authors:** A. F. M. Willich

**Affiliations:** London


### *On the practical Advantages of the Use of Flannel worn next the Skin, by Dr.* WILLICH.

IN conformity with the invitation held out in the Prospectus of this Journal, which is open to the investigation of every subject, in the various departments of medical science, if the matter under consideration be sufficiently interesting and deserving of notice, I presume to insert the following explanation.—I conceive it a duty incumbent on every author, to take the earliest opportunity to correct any ambiguities, or prevent misconstructions, to which any doctrines or statements in his publications may be liable, especially if the subject in question relate to matters of practical importance.

In my late work, intitled "*Lectures on Diet and Regimen,*" I have decidedly opposed and controverted the novel opinion, respecting *the use of flannel next the skin,* which has been maintained and spread, even in familiar life, among those who are hardly competent to judge for themselves, but implicitly rely on the advice of their medical friends. To such, therefore, and particularly the readers of my book beforementioned, I shall discharge difcharge an obligation which is no more than ftrictly due, by placing the difpute, or difference of opinion, on this popular fubject, in fuch a point of view, as will enable them to deduce a juft conclufion, whether or not the wearing of flannel, under particular circumftances, be ufeful or detrimental to health.

Having obferved in my "*Lectures*," p. 235, in the fection "*On the immediate Covering of the Skin*," that " not only analogy, but experience likewife proves, that wool worn next the fkin has indifputable advantages over all other fubftances;" and having ftated the reafons for which it ftands fo eminently recommended as a part of under-drefs, together with the good effects, and fuperior advantages with which the ufe of it is *generally* attended, I have proceeded to exprefs my furprize (p. 237), " that any individual, however great his reputation, fhould be whimfical or hardy enough to difpute matters of fact, merely with a view to eftablifh a favourite hypothefis."

Not inclined to retract a fingle word of this bold affertion, although I profefs the greateft veneration for the talents, learning and genius fo happily blended in the author of "*Zoonomia*," I fhall content myfelf (and herein, I truft, fatisfy my readers) with laying down the *conditions* and *modifications* in what relates to the ufe of this fubftance, as nearly as poffible, in the words of the celebrated Hufeland, the author of the "*Art of prolonging Human Life*;" a valuable work, of which the fecond edition has lately appeared at Jena, with confiderable additions and improvements.

As I have in no part of my Lectures recommended the ufe of flannel to children or young perfons, I do not hefitate candidly to quote this learned writer, even though his opinion fhould appear to vary in fome meafure from my own: for, as every medical man claims the privilege of determining agreeably to the notions formed by his reafon and experience, I may be permitted to differ from others, where I cannot pay homage to theory alone, and where I conceive there is a fufficient number of facts to warrant the juftice of my conclufions. Having previoufly defcribed the general properties and effects of flannel, Mr. Hufeland expreffes himfelf to the following purport: " Upon the whole, I am of opinion, that it would not be advifable, at leaft to children and young perfons, *univerfally* to adopt a woollen texture for the covering of the fkin; particularly as this drefs requires a more frequent change to promote cleanlinefs, and confequently would produce a contrary effect among the lower claffes of people*. It is, however, a falutary drefs to

* To anfwer this objection fatisfactorily, I beg leave to refer the reader to my arguments on the fame fubject, discuffed page 238, and foll. of the "*Lectures on Diet and Regimen*." W.

to those who, in all probability, have commenced the second half of their life; to all cold or phlegmatic temperaments; to all who lead a sedentary life; to individuals subject to catarrhs or frequent colds, gout, diarrhœa, and partial congestions of the blood; to all nervous patients and convalescents from severe chronic disorders; to persons who are too susceptible of the impressions of the atmosphere; and, lastly, in such climates and pursuits of life as are exposed to frequent and sudden changes of air. It is, on the contrary, hurtful * to those, without exception, who are already troubled with violent perspiration, or with cutaneous eruptions, and who cannot afford frequently to change their under-dress. Woollen drawers are highly pernicious to young persons."—And even in those cases where flannel next the skin is obviously conducive to health, Mr. Hufeland recommends only such a texture of wool as is sufficiently porous, and neither too rough nor too thick. Coarse woollen stockings in winter, and thin ones in summer, ought, in his opinion, to be more generally worn. Those persons, lastly, who are in a good state of health, and have no particular reason for wearing flannel, or whose skin is too irritable, may find it, he thinks beneficial, to wear a cloth fabricated of a mixed texture of cotton and linen.

*London, Feb.* 2, 1799. A. F. M. Willich.

* Mr. H. doubtless meant to say, that it is *then only* hurtful; if none of the external conditions before specified take place; which again render the wearing of flannel salutary to the individual, even in cases apparently dubious.

